# Giardiasis and Eosinophilia in Outpatients with Gastrointestinal Symptoms: A Case Series

**DOI:** 10.4269/ajtmh.24-0855err

**Published:** 2026-01-06

In the original research paper, “Giardiasis and Eosinophilia in Outpatients with Gastrointestinal Symptoms: A Case Series” by Laura Niño-Puerto et al., 2025, (https://doi.org/10.4269/ajtmh.24-0855), an error was introduced in the editing stages of the data reported prior to publishing. The dosage amount for metronidazole was incorrectly published in grams instead of milligrams. The correct dosage for the metronidazole treatment for the 26 patients with eosinophilia is 250 mg. The paper has since been corrected. The results and conclusions are unchanged. A summary of the corrected data is shown below:

“All outpatients diagnosed with giardiasis were treated with antiparasitic drugs. Twenty-six outpatients with eosinophilia received 250 mg of metronidazole, administered over 7 days, and four patients received a single 2 g dose of tinidazole.” (page 1019)

